# Supplementary material for: Research Progress on the Efficacy and Mechanism of Acupuncture in Treating Chronic Gastritis
Source: Diseases. 2025 Nov 7;13(11):363. doi: 10.3390/diseases13110363 (PMC12650923; doi:10.3390/diseases13110363)
Supplement: Supplementary file 1 [file diseases-13-00363-s001.zip › diseases-3916841-supplementary.pdf]

Combination therapy is used to treat CG.

Disease	Location	Center	Age(mean ±SD), y	Intervention	Acupoint	Time of intervention	Duration	N	Control			Measurement time points	Validated scale used	Objective Evaluation Criteria	Post-acupuncture changes compared to baseline	Adverse events	References
									Age(mean ±SD) , y	Treatment	N						
CNAG	CN	SC	42.37 ±6.3	TCM + Mox	BL21,BL23, RN12	Mox: QD, 20 min/point; TCM: QD, TID	8 weeks	100	37.21 ±7.7	TCM: QD	100	Before treatment, 8 weeks after treatment	TCM Body Constitution Chart	Infrared thermal imaging parameters: Skin temperature at RN12, BL20, BL23, and the GV; Serum IL-6 levels; Gastric mucosal	The treatment group achieved an overall response rate of 92%; Yang deficiency constitution showed significant improvement (P<0.05); Infrared thermography revealed increased mean skin temperatures at the RN12, BL20, BL23, and along the GV compared to pre-treatment levels (all P<0.05); Serum IL-6 levels decreased		[163]

														pathological indicators: Degree of inflammatory cell infiltration in the gastric mucosa.	significantly (P<0.05).	
CNAG	CN	SC	44.92 ±4.21	WM + AC + TCM	RN6,ST36,RN12,SP6,PC6	AC: QOD, 30 min; WM: QD, TID;	4 weeks	45	45.61 ±4.28	WM: oral	44	Before treatment, 4 weeks after treatment	TCMSS for Gastrointestinal Diseases	Serum inflammatory markers: TNF-α, IL-8, IL-1β, hs-CRP; Gastroscopy indicators: gastric mucosal	The total effective rate in the treatment group was 95.56%. TCMS showed significant improvement (all P<0.05). Serum inflammatory factors—TNF-α, IL-8, IL-1β, and hs-CRP—decreased significantly (all P<0.05). Gastroscopic indicators improved significantly	[162]

						TCM: QD								hyperemia, edema, bile reflux, and incidence of erosion; Helicobacter pylori (Hp) eradication rate.	(all P<0.05). The Hp eradication rate reached 88.89%.		
CNAG	CN	SC	41.8 ±13.4	TCM + AA + WM	RN12,BL21, BL17,RN8,BL21	AA: QD, 6h; WM: oral	2 weeks	110	42.2 ±12.7	WM + AA: as per protocol	107	Before treatment, after treatment, 1-month follow-up	Symptom scoring	Hp seroconversion rate, gastrointestinal hormones, inflammatory factors	Patients with reduced TCMSS exhibited significantly elevated levels of gastrin and motilin, with a Hp seroconversion rate of 93.64%. Serum TNF-α and IL-6 levels decreased markedly, while PGE2 levels increased.	Observation group: 6 cases of mild skin reactions, resolv	[157]

																ed after symptomatic treatment.	
CNAG	CN	SC	46.31 ±10.45	AA + WM	RN12,ST36, BL23	AA: QD, 5h; WM: BID	3 weeks	60	47.15 ±11.22	WM: BID	60	Before treatment, after treatment	TCMSS Table for Gastrointestinal Diseases Chinese Medicine Diagnosis, Treatment Effectiveness Standards for Diseases and Syndromes	Serum motilin (MTL), gastrin (GAS), and somatostatin (SS) levels	The total effective rate was 95%, significantly higher than the control group (80%); scores for symptoms such as pantosade and epigastric pain decreased and were superior to the control group; GAS levels decreased while MTL and SS levels increased, P<0.05.		[158]
C	C	S	45.92	TCM	RN12,ST36,	TC	6	6	47.68	TCM:	6	Before	Diagnosti	Gastrosc	The overall effective	The	[159]

NAG	N	C	±5.61	+ AA	BL23	M: BID	weeks	0	±6.03	oral	0	treatment, after treatment	c Criteria and Therapeutic Efficacy Standards for Traditional Chinese Medicine Conditions	opic mucosal findings (erosion, hyperemia, edema) Serum gastrointestinal hormones (MTL, GAS, SS)	rate was 88.3%, significantly higher than the 70.0% in the control group (P<0.05). Post-treatment, GAS decreased while MTL and SS increased, with the observation group showing superior improvement compared to the control group (P<0.05). Gastroscopic examination revealed markedly reduced mucosal inflammation.	incidence of adverse reactions in the observation group was 8.33%, while that in the control group was 6.67%. The difference between the

																two groups was not statistically significant (P > 0.05).	
CNAG	CN	SC	34.12 ±4.22	TCM + AA	BL21,ST36,RN12	TCM: QD, BID; AA: QD, 2h	2.43weeks	40	36.23 ±3.54	TCM: oral	40	Before treatment, after treatment, one month after discontinuing medication	Guidelines for Clinical Research of New Traditional Chinese Medicine Drugs,TCMSSTable for Gastrointestinal Diseases, Consensus Statement	Clinical symptom score, H. pylori eradication rate	The overall effective rate was 85.00%, higher than the 62.50% in the control group. Symptom scores decreased significantly after treatment and were superior to those in the control group (P<0.01). The Hp eradication rate was 87.50%, higher than the 65.00% in the control group (P<0.01).		[184]

													on the Diagnosis and Treatment of Chronic Gastritis Using Integrated Traditional Chinese and Western Medicine Expert Consensus on the Diagnosis and Treatment of Chronic Gastritis in Traditional Chinese				
CNAG	CN	SC	40.59±3.38	AC + ACE	AC:EX-B3,BL18,BL19,BL21,BL21；ACE:RN12,RN10,BL18,BL21,BL21,ST36,RN6	AC: TIW; ACE: Q2W	6 weeks	64	39.97±3.97	AC: PC6,ST36,RN12,SP4 + SP9,RN4	64	Before treatment, after treatment		Gastroscopy score, gastric mucosal blood flow,	Overall efficacy rate: 90.63% (P=0.032 < 0.05); The treatment group exhibited lower scores for TCM syndrome patterns and gastroscopy findings, along with higher SF-36 scores and gastric mucosal blood flow (P<0.05 or P<0.001).		[164]

													Medicine, TCMSS, clinical efficacy, SF-36 questionnaire			
CNAG	CN	SC	57.4 ±7.6	TCM + AC + ACE	AC:ST36,ST25,RN12,RN10,RN4,LI10,SP6,BL21,BL25,SP10,RN6;ACE:ST25,RN4,RN10,KI16	TCM: QD, BID, d1-6; AC: QD, d1-6; ACE: QW	24 weeks	48	57.4 ±8.1	WM: TID	48	Before treatment, after treatment	Consensus on Diagnosis and Treatment of Chronic Atrophic Gastritis in TCM (2009, Shenzhen)，Chinese Consensus on Chronic Gastritis，Guideline	Gastric Mucosal Pathological Grading (Atrophy, Intestinal Metaplasia, Dysplasia) Results of Electronic Gastroscopy	The treatment group showed a significant reduction in clinical symptom scores (P<0.05), accompanied by decreased gastric mucosal atrophy, intestinal metaplasia (P<0.001), and lower pathological grading of dysplasia (P<0.05).	[185]

													s for Clinical Research on New Traditional Chinese Medicines (Trial) Clinical Symptom Scoring				
CNAG	CN	SC	35.9	ACE + WM	ST36, RN12, PC6;	WM: BID; ACE: Q2W	15.43 weeks	100	39.1	WM: BID, 2w	100	Before treatment, after treatment	Guidelines for Clinical Research of New Traditional Chinese Medicines	Clinical symptom scores, gastroscopy and histopathological biopsy results, incidence of adverse reactions	Overall clinical response rate: 89% ($P < 0.05$); Endoscopic response rate: 88.00%,	Adverse reactions occurred in 13 individuals.	[161]
C	C	S	42.30	TCM	BL21,BL21,S	TC	4	5	42.31	WM:	5	Before	Expert	TCMSS,	Overall efficacy rate:		[160]

NAG	N	C	±2.14	+ ACE	T36,ST44,LR3,SP6	M: QD, BID; ACE: every 10d	weeks	0	±2.12	BID, 1m	0	treatment, after treatment, and at the 6-month follow-up	Consensus on the Diagnosis and Treatment of Chronic Gastritis in Traditional Chinese Medicine (2017)	PGI, PGII, PGI/PGII, IL-32, CGRP, EGF, clinical efficacy, incidence of adverse reactions, incidence of atrophic gastritis, 6-month recurrence rate	98.00% (vs. control group 84.00%, P<0.05); Superior improvement in multiple indicators (P<0.05); Low incidence of adverse reactions; Incidence and recurrence rate of atrophic gastritis both 0% (P<0.05).	
CNAG	CN	SC	18~60	TCM + ACE	RN6,RN4,RN12,BL18,BL21,BL21,BL17,BL23,SP6,ST36	TCM: QD, TI	12 wee	31	18~60	TCM: oral; ACE: as protocol; WM:	Chinese M	Before treatment, after treatment	TCMSS for Gastrointestinal Diseases	Degree of improvement in gastric	Overall efficacy rate: 93.54%, pathological efficacy rate: 87.09% (P<0.05); Significant reductions in clinical	[186]

	D; ACE: QW	ks	TID	edicine Group 30 Acupuncture Thread Implantata	, Clinical Symptom Scoring from the Chinese Consensus on Chronic Gastritis (2017)	mucosal pathology<br>Overall pathological response rate<br>Meridian infrared skin temperature	symptom scores, gastric mucosal atrophy, and intestinal metaplasia, along with increased meridian infrared skin temperature ($P<0.05$).

											tion Group 31 Western Medicine Group 32					
CN	CN	SC	53.82 ±6.3	WM + TCM	RN12,BL21, ST36	WM:	12	66	53.70 ±6.2	WM: BID	66	Before treatment,	Interpretation of	Endoscopic	The recent efficacy rate reached 90.91%,	[187]

AG			4	+ ACE		QOD; TCM: QD, BID; AA: QW	weeks		9			after treatment	Clinical Diagnosis and Treatment Guidelines for Traditional Chinese Medicine (Spleen and Stomach Disorders Volume) TCMSS System	pathological grading, inflammation-related factors, gastrointestinal hormones, cellular immune function, H. pylori eradication rate, adverse reaction incidence	with significant reductions in TCMSS and endoscopic pathological scores. Hp eradication rates were higher than the control group (P<0.05). Post-treatment levels of inflammation-related factors decreased significantly (P<0.05), while gastrointestinal hormone indicators increased (P>0.05). Cellular immune function indicators also showed elevated levels (P>0.05).		
CNAG	CN	SC	49.59 ±4.25	TCM + ACE + WM	ST25,RN4,RN10,KI16	WM: oral; TC	4 week	53	49.52 ±4.23	WM: QD+TID+BID, 2w	53	Before treatment, after treatment, 1-year	Guidelines for Diagnosis and Treatment	HP Eradication Rate	Overall efficacy rate: 98.11%, HP eradication rate: 96.23%. TCMSS improved in	Adverse reactions occurr	[188]

						M: QD; ACE: Q2W	s					follow-up	of Digestive Diseases in TCM Consensus Opinions from the National Symposium on Chronic Gastritis TCMSS System		all groups (P<0.05 or P<0.01); Recurrence rate: 13.21%.	ed in 2 individuals.	
CNAG	CN	SC	51.79 ±6.20	TCM + ACE + WM	RN12,BL21, ST36	WM: oral; TCM: QD, BID; AA:	2 weeks	60	51.86 ±6.23	WM: BID+TID+BID, 2w	60	Before treatment, after treatment	Guidelines for Clinical Research on New TCM Consensus on Chronic Gastritis Consensus on	Pathological scoring, inflammatory cytokines, gastrointestinal hormones,	The overall effective rate was 95.00%. Scores for gastric mucosal inflammation, inflammatory activity, glandular and intestinal metaplasia, as well as serum levels of IL-8, IL-11, and TNF-α were significantly reduced (P < 0.05 for all). Meanwhile, GAS and	Both groups demonstrated low rates of adverse reactions	[189]

						QW							Integrated Traditional Chinese and Western Medicine Diagnosis and Treatment of Chronic Gastritis (2011) Symptom and Sign Scoring		SS levels were significantly elevated (P < 0.05 for both).	and good safety profiles.	
CAG	CN	MC	52.43 ±8.29	WM + AC + TCM	BL21,ST36,PC6,BL21,RN12,PC3	AC: QOD, 20-30 min; TCM:	4 weeks	39	52.43 ±8.29	WM + AC	39	1 day before treatment, 4 weeks after treatment	TCMSS	Serum PGI, PGII, serum interleukin-17 (IL-17) levels, and gastrosc	The overall response rate in the treatment group was 92.31%. Both PGI and PGII levels increased significantly compared to pre-treatment levels (P<0.05), while serum IL-17 levels decreased significantly (P<0.05).	The treatment group reported 2 cases of nausea and	[165]

						BID; WM: BID								opic assessment of gastric mucosal status	TCMSS showed a significant reduction (P<0.05).	vomiting, 1 case of headache, and 1 case of diarrhea, with an adverse reaction incidence rate of 10.26%.	
CAG	CN	SC	37~78	TCM + AC	RN12,ST36	AC: every	12we	15	Control Group 1:	TCM: oral; WM: oral	15 per	15 days, 3 months	Clinical Syndrome Scoring Table for	Pathological biopsy, H. pylori	The overall response rate in the treatment group was 92.9%; chronic inflammation		[190]

						3-4d; TCM: QD, BID	eks		35–76; Control Group 2: 35–76		group		the “Guidance Principles for Clinical Research on New Traditional Chinese Medicines”	eradication status, or grading status	showed significant improvement (P<0.05); Hp eradication rate reached 87.5%; IM and ATP were reversed.	
CAG	CN	SC	41.06 ±6.70	WM + TCM + AC	SP6,RN4,RN12,ST25,PC6,ST36	AC: QOD, 20 min; Mox: QOD, 20 min; TC	12 weeks	43	41.38 ±6.34	WM: oral	43	Before treatment, 3 months after treatment	Consensus Guidelines for the Diagnosis and Treatment of Chronic Atrophic Gastritis Using Integrated Traditional Chinese and	Pathological changes of chronic inflammation, intestinal metaplasia, and atrophy observed during gastrosc	The overall response rate in the treatment group was 86.05%. Post-treatment, the accumulation of pathological signs was significantly reduced. Chronic inflammation, intestinal metaplasia, and atrophy showed marked improvement. Post-treatment, the expression of Wnt3 and β-catenin in gastric mucosa, along with	[191]

		M: BID; WM: TID, QD		Western Medicine (2017) Syndrome Scoring Criteria	opy; expression of Wnt3 and β-catenin in gastric mucosa; serum levels of transforming growth factor-alpha (TGF-α), basic fibroblast growth factor (bFGF), and chemokine 10 (CXCL1	serum levels of TGF-α, bFGF, and CXCL10, were significantly decreased (P<0.05).

														0).			
CAG	CN	SC	52.54 ±3.6	AC + TCM	RN4,RN8,RN12,RN9,ST22,SP6,ST36,ST25,RN6	Mox: as per procedure; TCM: QD, BID	12 weeks	35	52.23 ±4.0	WM: oral	35	Before treatment, 3 months after treatment	Chinese Medicine Symptom Rating Scale for Gastrointestinal Diseases	Gastroscopy findings: gastric mucosal atrophy, intestinal metaplasia, dysplasia; Routine laboratory tests, liver function tests, renal function tests, electrocardiogram.	The overall response rate in the treatment group was 94.29%. TCMSS decreased significantly after treatment (P<0.05). Pathological scores for glandular atrophy, dysplasia, and intestinal metaplasia improved significantly after treatment (P<0.05).	During treatment and follow-up, no significant adverse reactions were observed in either group. Routine laboratory tests, liver	[192]

																function tests, renal function tests, and electrocardiograms showed no abnormalities.	
CAG	CN	SC	49.54 ±4.83	WM + TCM + AC	BL21, RN12, PC6, ST36, PC3	TCM: TID; AC: QD, 30	8 weeks	43	49.75 ±5.02	WM: oral	43	Before treatment, 4 weeks after treatment, 8 weeks after treatment	None	Pathological scores for gastric mucosal atrophy, active/chronic	The overall response rate in the treatment group was 95.35%. The scores for primary and secondary TCM syndromes, as well as the total TCMSS, significantly decreased compared to pre-	Two cases of mild diarrhea occurred in the	[167]

	min, 5/wk; WM: for 1-2w		inflammation, intestinal metaplasia, and dysplasia during gastroscopy; Hp conversion rate; Inflammatory markers [Neutrophil-to-lymphocyte ratio (NLR), Interleukin-1β (IL-1β)]; Gastric mucosal	treatment levels (P<0.05). Pathological scores for gastric mucosal atrophy, active inflammation, chronic inflammation, intestinal metaplasia, and dysplasia significantly decreased compared to pre-treatment levels (P<0.05). Hp conversion rate was 90.70%; NLR and IL-1β levels were significantly reduced compared to pre-treatment (P<0.05); PGI and PGI/PGII levels were significantly increased compared to pre-treatment (P<0.05); G-17 levels at 4 and 8 weeks post-treatment were significantly	treatment group, with an incidence rate of 4.65%.

														function markers [Pepsinogen I (PGI), PGI/PGII ratio, Gastrin-17 (G-17)]	elevated compared to pre-treatment and showed a sustained upward trend (both P<0.05).		
CAG	CN	SC	45.3 ±4.5	TCM + AC	SP3,SP4,SP8	AC: QD, 15 min; TCM: QD, BID	3 weeks	62	44.5 ±4.2	WM: oral	62	Before treatment, 21 days after treatment	Semi-quantitative scoring method (symptoms, signs, triggers, work capacity, daily living capacity)	Serum EGF levels; Serum nitric oxide (NO) levels	The overall response rate in the treatment group was 88.71%; serum EGF levels decreased compared to pre-treatment levels (P<0.05); serum NO levels increased compared to pre-treatment levels (P<0.05).		[193]
CAG	CN	MC	55.5 ±10.1	TCM + AC	ST36, ST34, SP4, PC6, RN12	TCM: BI	6 we	64	53.1 ±10.8	WM: oral	62	Before treatment, 6 weeks after	Patient-Reported Outcomes	Gastric Mucosal Patholog	The overall response rate in the treatment group was 71.88%.	One advers e	[194]

	D; AC: QD	eks		treatment	(PRO) Scales	y Score (Chronic Inflammation, Active Inflammation, Atrophy, Intestinal Metaplasia, Dysplasia)	Pathological scores for chronic inflammation, atrophy, and intestinal metaplasia in gastric mucosa showed significant reduction compared to pre-treatment levels (all P<0.05), while scores for active inflammation and dysplasia also decreased. The reversal rates for OLGA and OLGIM were 70.31% and 79.69%, respectively. Clinical symptoms showed significant reductions in epigastric fullness and epigastric pain scores compared to pre-treatment (all P<0.05). The PRO questionnaire revealed significant decreases in	event (1.56%) and two drug-related adverse reactions (3.13%) occurred in the treatment group.

															acid reflux, dyspepsia, bowel movements, psychological state, systemic symptoms, and total scores compared to pre-treatment (all P<0.05).	
CAG	CN	SC	51±8	AC + WM	RN12,PC6,ST36	AC: QOD, 30 min; WM: BID	2 weeks	67	52±7	WM: oral	67	Before treatment, after treatment	None	Serum interleukin-17 (IL-17) levels, serum interleukin-10 (IL-10) levels, H. pylori eradication rate (14C urea breath test)	The total response rate in the treatment group reached 94.0%. Scores for abdominal distension, epigastric pain, rumbling, heartburn, acid regurgitation, and poor appetite were significantly lower than pre-treatment levels (P<0.05). Serum IL-10 levels were significantly higher than pre-treatment levels (P<0.05), while serum IL-17 levels were significantly lower than pre-	[195]

															treatment levels ($P<0.05$). Hp eradication rate reached 92.5%.	
CAG	CN	SC	40.68 ±9.6	TCM + AC	RN12,SP4,PC6,ST36,BL21	AC: QD, 15 min, d1-7 then 3d off; TCM: QD, BID	4 weeks	60	39.46 ±7.9	WM: oral	60	Before treatment, one month after treatment	TCMSS Table for Gastrointestinal Diseases	Gastroscopic indicators: Number of gastric mucosal erosions, hyperemia, and hemorrhagic spots; Gastric mucosal histological indicators: Degree of	The total effective rate in the treatment group was 83.33%. Scores for gastric pain, bloating, burning sensation with refusal of pressure, and belching with acid reflux significantly decreased compared to pre-treatment levels ($P<0.05$), while scores for reduced appetite showed no significant change. The number of gastric mucosal erosions, hyperemia, and hemorrhagic spots significantly decreased compared to pre-treatment levels ($P<0.01$), with a	[196]

														epithelial erosion repair and improvement in inflammatory cell infiltration;	45.00% rate of apparent improvement in gastric mucosal inflammation. Histological recovery rate was 45.0%, with restoration of eroded gastric mucosal epithelium and improvement in inflammatory cell infiltration.		
CAG	CN	SC	51.29 ±6.26	WM + TCM + AC	BL21, BL21, RN12, ST36, PC6	AC: QD, 30 min; TCM: QD, BID; W	8 weeks	69	51.24 ±6.23	WM: oral	69	Before treatment, 2 months after treatment, 6 months after treatment, 1 year after treatment	TCMSS	Serum inflammatory factors: Interleukin-6 (IL-6), Tumor Necrosis Factor-β (TNF-β) levels; Gastroscopy	The overall response rate in the treatment group was 98.55%; symptom scores showed a significant reduction compared to pre-treatment levels (P<0.05); 3. Serum IL-6 and TNF-β levels both decreased significantly compared to pre-treatment levels (P<0.05); The recurrence rate at 6	Incidence rate: 2.90%, including 1 case of fatigue (1.45%) and 1	[197]

						M: 3-4 times daily, TID								indicators: Improvement in gastric mucosal inflammation, glandular atrophy, and intestinal metaplasia.	months post-treatment was 0.00%, and the 1-year recurrence rate was 1.45%.	case of gastrointestinal reaction (1.45%)	
CAG	CN	SC	40±2.1	WM + TCM + AC	ST36	TCM: QD	12 weeks	32	41±2.3	WM + WM	32	Before treatment, 12 weeks after treatment	Guidelines for Clinical Research on New Traditional Chinese Medicines: TCMSS Table	Gastroscopy-guided biopsy (MTB) assessment criteria: Improvement in	The overall response rate in the treatment group was 93.8%. Scores for primary symptoms such as epigastric fullness, epigastric distension and pain, and epigastric discomfort showed significant		[198]

														gastric mucosal inflammation, glandular atrophy, intestinal metaplasia, and dysplasia	reduction compared to pre-treatment levels (P<0.05). Pathological changes including gastric mucosal inflammation and glandular atrophy improved compared to pre-treatment findings.	
CAG	CN	SC	60±4.56	AC + WM	ST42,SP4	AC: QD, 50 min; WM: TID	3 weeks	30	60±4.56	WM: oral	30	Before treatment, 90 days after treatment	TCMSS Scale	Gastric Mucosal Changes Observed During Gastroscopy	The overall response rate in the treatment group reached 90.00%. The total clinical symptom and sign scores decreased significantly, with marked improvement in symptoms including epigastric pain, loss of appetite, abdominal distension, loose stools, and limb	[199]

															weakness. Gastroscopy revealed significant improvement in gastric mucosal condition.	
CAG	CN	SC	51.34 ±3.7	AC + TCM	ST34	AC: QD, 20 min; TCM: QD, BID	12 weeks	35	52.27 ±4.1	WM: oral	35	Before treatment, 3 months after treatment	TCMSS Scale	Gastroscopic changes in gastric mucosa and pathological examination (glandular atrophy, dysplasia, intestinal metaplasia)	The total effective rate in the treatment group reached 91.43%; TCMSS showed significant reduction (P<0.05); pathological scores demonstrated marked improvement, with reductions in glandular atrophy, dysplasia, and intestinal metaplasia (P<0.05).	[200]
CA	CN	SC	48.15 ±12.	WM + TCM	SP6,ST36,PC6,ST37,ST39,	AC:	12	60	48.60 ±11.7	WM + TCM	60	Before treatment, 4	TCMSS Scale	Histological	The overall response rate in the treatment	[201]

G			90	+ AC	ST25,RN12, RN4	QD, 30 min; TCM: QD, BID; WM: BID	weeks		8			months after treatment		indicators during gastroscopy (glandular atrophy, intestinal metaplasia, chronic inflammation, dysplasia, lesion activity)	group reached 96.67%. TCMSS showed a significant decrease, with all scores significantly lower than pre-treatment levels (P<0.05). Gastroscopic histological indicators demonstrated marked improvement: post-treatment scores for glandular atrophy, intestinal metaplasia, chronic inflammation, dysplasia, and lesion activity were all significantly lower than pre-treatment levels (P<0.05).		
CAG	CN	SC	40±7	TCM + AC + Mox	BL21, PC6, RN12, ST36, SP4	AC: QD, 30 min	4 weeks	75	40±7	TCM: oral	75	Before treatment, 28 days after treatment	TCMSS Scale	Healing status of erosions observed during gastrosc	The total effective rate in the treatment group was 92.0%. TCMSS showed a significant reduction (P<0.05). The number of erosive	Four cases of diarrhea, two	[166]

						; TCM: TID								opy; Serum levels of inflammatory cytokine IL-6	lesions observed during gastroscopy decreased markedly (P<0.05). Serum IL-6 levels decreased significantly (P<0.05).	cases of rash, and two cases of needle phobia occurred, with an adverse reaction rate of 10.7%.	
CAG	CN	SC	58.64 ±2.12	WM + TCM + AC	BL21, RN6, ST36, SP4, RN10	AC: QOD, 20	8 week	49	58.61 ±2.10	WM: oral	51	Before treatment, 2 months after treatment	TCMSS Scale	Gastroscopic indicators: intestina	The total effective rate in the treatment group was 95.92%; improvement in TCMSS (P<0.05);	One case of headache	[202]

	min; TCM: QD; WM: 3-4 times daily, TID	s	l metaplasia grade, gastric mucosal characteristics, gastric atrophy grade; Serum gastrointestinal hormone indicators: motilin, somatostatin, gastrin; Serum inflammatory factors: IL-6, IL-8, TNF-	improvement in gastroscopic indicators (P<0.05); serum gastrointestinal hormones: significant increase in motilin, significant increase in SS, and significant decrease in gastrin (all P<0.05); Serum inflammatory factors: IL-6, IL-8, and TNF-α levels significantly decreased (all P<0.05); Hp positivity rate significantly decreased (P<0.05); Immune function: CD_4^+ significantly increased, CD_8^+ significantly decreased, and CD_4^+/CD_8^+ ratio significantly increased (all P<0.05).	and one case of rash occurred, with an adverse reaction incidence rate of 4.08%.

														α; Hp positivity rate; Immune function indicators: T lymphocyte subsets		
CAG	CN	SC	47.87±8.81	TCM + Mox	RN12,ST36,ST25,LI11	Mox: QD, 20 min/point; TCM: QD	8 weeks	32	47.87±8.81	TCM: oral	32	Before treatment, 60 days after treatment	TCMSS Scale	Pathological indicators during gastroscopy: Degree of atrophy in gastric mucosal epithelium and glands, number	The overall response rate in the treatment group was 87.50%. TCMSS showed a significant reduction compared to pre-treatment levels (P<0.05). Gastroscopic findings revealed: reduced gastric mucosal atrophy, increased glandular number, improved mucosal basal layer thickening, and alleviation of pyloric	[169]

												of glands, thickness of the basal layer of the mucosa, presence of pyloric gland metaplasia/intestinal metaplasia.	glandular metaplasia/intestinal metaplasia.		
CAG	CN	SC	53.23±9.96	TCM + Mox	RN12,RN6,RN8,ST36	Mox: QD, 30 min; TCM:	12 weeks	30	55.63±10.24	WM: oral	30	Before treatment, 12 weeks after treatment	Patient-Reported Outcomes (PRO) Scales	Gastroscopy and pathological indicators: Degree of gastric	The total effective rate in the treatment group was 90.0%. Endoscopic examination revealed reduced gastric mucosal whiteness, diminished vascular exposure, and	One case showed a slight decrease in serum potass	[170]

	TID		mucosal atrophy, gastric mucosal pathological score; Serum inflammatory factors: Levels of TNF-β, IL-1β, IL-4, IL-10, IL-37, TGF-β; Molecular biology indicators: Relative expression levels	improvement in mucosal erythema and hemorrhagic spots. Pathological scores for gastric mucosal lesions showed significant improvement (all $P<0.05$). PRO questionnaire scores decreased significantly (all $P<0.05$). Serum inflammatory factors: TNF-β, IL-1β, IL-37, and TGF-β levels significantly decreased, while IL-4 and IL-10 levels significantly increased (all $P<0.05$); Molecular biology indicators: Relative expression levels of STAT3 mRNA, STAT3, p-STAT3, mTOR, and p-mTOR significantly decreased	ium levels.

														of STAT3 and mTOR mRNA in gastric mucosa; Relative expression levels of STAT3, p-STAT3, mTOR, and p-mTOR proteins.	(all P<0.05).	
CAG	CN	SC	41.56 ±3.73	TCM + AC + WM	PC6, RN12, ST36, ST21, RN10	AC: QD, 30 min;	12 weeks	59	40.38 ±4.15	WM: oral	59	Before treatment, 3 months after treatment	TCMSS Scale	Gastroscopic indicators: mucosal color, mucosal	The total effective rate in the treatment group was 93.22%; Significant improvement in TCM syndromes (all P<0.05); Significant	[203]

	WM: BID, TID; TCM: QD, BID		texture, mucosal folds, mucosal granularity, vascular transparency score; Pathological indicators: chronic inflammation, inflammatory activity, glandular atrophy, intestinal metaplas	reduction in gastroscopic scores (all $P<0.05$); Significant reduction in pathological scores (all $P<0.05$); Serum markers: Significant decrease in NF-κB, TNF-α, COX-2, and EGF levels (all $P<0.05$); Hp conversion rate to negative: 94.92%.

														ia, dysplasia score; Serum indicators: NF-κB, COX-2, TNF-α, EGF levels; Hp conversion rate		
CAG	CN	SC	47.25±7.63	WM + TCM	BL21,LR13,PC6,SP4,ST36,RN12	WA: QOD; TCM: QD, BID; WM:	8 weeks	45	46.81±7.96	WM: oral	45	Before treatment, 8 weeks after treatment	TCMSS Scale	Vascular endothelial growth factor markers: Serum EGF, VEGF, BFGF; Pathological	The overall response rate in the treatment group was 95.56%. Vascular endothelial growth factor (VEGF) and basic fibroblast growth factor (BFGF) levels significantly decreased, while EGF levels significantly increased (all $P < 0.05$). Pathological	[204]

						BID, TID							markers: Gastric mucosal atrophy, intestinal metaplasia, dysplasia grading	scores significantly decreased (all P < 0.05). TCMSS significantly decreased (all P < 0.05).	
CAG	CN	SC	20~63	TCM + WM	ST36,PC6,RN12	WA: QD; TCM: QD	16 weeks	40	21~63	WM: oral	40	Before treatment, 4 months after treatment	Gastroscopic pathological indicators: Gastric mucosal inflammation, glandular atrophy, intestinal	The total effective rate in the treatment group was 92.5%; gastroscopic pathological scores showed a significant decrease (all P<0.05); serum pepsinogen levels: PGI and PGR showed a significant increase (both P<0.05), while PGII showed no significant change.	[205]

														metaplasia, dysplasia grading; Serum pepsinogen indicators: PGI, PGII, PGR (PGI/PGII) levels; Hp detection		
CAG	CN	SC	56±8	AC + AA	BL21,ST36,RN6,RN10,SP4	Therapy: QOD, 20 min	8 weeks	45	58±8	WM: oral	45	Before and after treatment	Efficacy is determined in accordance with the Diagnostic and Therapeut	Gastroscopy: Degree of gastric atrophy, gastric mucosal	The overall response rate in the observation group was 97.8%. Clinical symptom scores and total scores decreased, with reduced disease severity, improved	[172]

													ic Effect Standards for Traditional Chinese Medicine Diseases and Syndromes.	characteristics, degree of intestinal metaplasia; Serum inflammatory factors: Levels of TNF-α, IL-6, IL-8	gastric mucosal characteristics, lower intestinal metaplasia rates, significantly reduced serum TNF-α, IL-6, and IL-8 levels, decreased serum gastrin levels, and increased serum SS and MTL levels. $P<0.05$.	
CAG	CN	SC	52.30±10.76	TCM + AA	RN12,BL21, ST36,PC6	Therapy: QD, 2h	12 weeks	56	51.73±11.06	WM: TID	57	Before treatment, 1 week, 2 weeks, 4 weeks, after treatment	TCMSS	Gastroscopic mucosal findings and histopathological scoring	The overall response rate in the treatment group was 78.6%, with all scores decreasing compared to pre-treatment levels; scores for mucosal atrophy and intestinal metaplasia were lower than pre-treatment	[173]

															levels.	
CAG	CN	SC	54.87±11.22	TCM + AA	AA:ST36,BL21,RN4,RN12	TCM: QD, BID	24 weeks	30	57.03±12.50 59.77±9.39	TCM: oral	Reference 1: 31 Reference 2: 30	Day 1 of treatment and 1, 2, 3, 4, 5, and 6 months after treatment	Developed in reference to the 2002 edition of the "Guidelines for Clinical Research on New Traditional Chinese Medicines"	Fasting serum or plasma: PGI, PG I/II	Effective rate: 40%. PGI and PGI/II levels increased (P < 0.05).	[206]
CAG	CN	SC	54.87±11.22	TCM + AA	RN12,BL21, ST36	TCM: QD, BID; AA: as per	24 weeks	30	1 57.03±12.50；2 59.77±9.39	WM: TID;TCM: oral; WM: TID	Reference 1: 31; R	Before treatment, Day 1 post-treatment, Months 1, 2, 3, 4, and 6 post-treatment	Guidelines for Clinical Research on New Traditional Chinese Medicines Consensus	Gastroscopy indicators (gastric mucosal color), pathological	TCMSS improved, with significant reductions in the total pathological score of the lesser curvature of the gastric antrum, gastric angle, and greater curvature of the gastric body, as well as	[171]

						course				eference 2:30		Opinion on Traditional Chinese Medicine Diagnosis and Treatment of Chronic Atrophic Gastritis TCMSS	indicators (total pathological score for the lesser curvature of the gastric antrum, gastric angle, greater curvature of the gastric body, etc., atrophy score)	the atrophy score of the lesser curvature of the gastric antrum (P<0.05).		
CAG	CN	SC	34.12 ±4.22	AA + TCM	BL21,ST36,RN12	TCM: QD , BI	40	36.23 ±3.54	TCM: QD, BID	40	Before treatment, after treatment, one month	Guidelines for Clinical Research on New	Clinical symptom score, H. pylori eradicati	The overall effective rate was 85.00%, significantly higher than the 62.50% in the control group.	.	[175]

						D; AA : QD , 2h						after discontinuing medication	Traditional Chinese Medicines Traditional Chinese Medicine Syndrome Scoring Table for Gastrointestinal Diseases Consensus on Integrated Traditional and Western Medicine Diagnosis and Treatment of Chronic Gastritis	on rate (detected by 13C urea breath test)	Symptom scores decreased significantly after treatment and were superior to those in the control group (P<0.01). The Hp eradication rate was 87.50%, higher than the 65.00% in the control group (P<0.01).		
C	C	S	43.9	ACE	BL21,RN12,	W	1	7	45.2	WM +	6	Before	Diagnosti	TCMSS,	The overall response	.	[174]

AG	N	C	±4.7		ST36	M: for 2w; ACE: QW	2 weeks	1	±4.8	TCM: TID, 3m	8	treatment, 1 day after treatment, 6 months after treatment	c and Therapeutic Effectiveness Standards for Traditional Chinese Medicine Syndromes, PRO Scales, HAMA Scales	gastroscopic mucosal grading, histopathological grading, H. pylori eradication rate and recurrence rate, serum G-17, PGI, PGII, PGI/PGII ratio	rate in the treatment group was 98.59%. The TCMSS, gastric mucosal mottling/erosion score, pathological chronic inflammatory response/activity score, and HAMA scale score all decreased compared to pre-treatment levels (P<0.01). Serum G-17, PGI, PGII levels, and PGI/PGII ratio all increased (P<0.01, P<0.05). The recurrence rate was 2.99%.		
CG	CN	SC	49.51±4.31	WM + AC + Mox + TCM	ST44,SP4,ST36,SP6,RN12,RN4,RN6,PC6,DU20,EX-HN1,SP15	AC: QD, 30 min	8 weeks	32	49.52±3.97	WM + AC + Mox	32	Before treatment, after 8 treatment cycles	TCMSS Scale	Gastroscopic assessment of gastric mucosal	The overall response rate in the treatment group was 96.88%. Gastric function indicators showed significant	One case of nausea and two	[207]

						; TCM: QD								condition (improvement in band-like or patchy erythema); Serum gastric function markers: GAS, MTL, Substance P (SP) levels	improvement (all P<0.05). Endoscopic examination revealed marked improvement in band-like or patchy erythema of the gastric mucosa.	cases of vomiting occurred, with an adverse reaction incidence rate of 9.38%.	
CG	CN	SC	20~70	Mox + TCM	ST36,PC6,ST25,RN13,RN12,RN10,RN6	Mox: QD, 25-35 min;	4 weeks	45	20~70	WM: oral	45	Before treatment, 30 days after treatment	Visual Quantification Scorecard	Gastric mucosal status under endoscopy (edema, exudatio	The overall response rate in the treatment group was 88.9%. Gastroscopy findings showed significantly improved efficacy. Gastric pain was alleviated, with a		[208]

						TCM: QD, BID								n, mucosal fold morphology, lymphocyte and plasma cell infiltration); Hp test results	significant decrease in the proportion of severe pain compared to pre-treatment levels, and a significant increase in the proportion of mild pain and no pain. The Hp conversion rate improved, gastric mucosal edema and exudation disappeared, mucosal folds largely returned to normal, and lymphocyte and plasma cell infiltration significantly decreased.	
CG	CN	SC	52.53 ±12. 95	Mox + AA	Ai-Box Moxibustion Acupoint Selection: Group 1 (RN12, RN8, ST36, SP6) Group 2	Mox: QD, 20 min; AA	2 weeks	32	Ai Box Moxibustion Group: 54.00	Mox: QD; AA: QD	32 in each grou	Before treatment, 14 days after treatment	Chinese Medicine Syndrome Rating Scale for Gastrointestinal Diseases	None	The total effective rate in the treatment group was 90.6%; TCMSS showed significant improvement (all $P<0.05$); VAS scores decreased significantly compared to pre-	[209]

					(BL21, DU14, KI1)	: QD, 20 min			± 11.10; Chinese Herbal Medicine Warming External Application Group: 54.09 ± 11.00		p		Visual Analogue Scale (VAS) for Pain		treatment levels (P<0.05).	
CG	CN	SC	37.6 ±16.9	AC + TCM	RN12, PC6, ST36, LR3, ST44, ST39	AC: QD, 30	4 week	40	39.1 ±17.6	WM: oral	40	Before treatment, 4 weeks after treatment, 6 months	None	Gastroscopy: Signs of gastric mucosal	The overall response rate in the treatment group was 92.5%. Long-term efficacy (six months after	[210]

						min; TCM: QD, BID	s					after discontinuation		inflammation	discontinuation): Recurrence rate: 12.5%, with sustained improvement in symptoms.	
CG	CN	SC	59.05 ±15.42	WM + AC + AP	Auricular points: Spleen, Stomach, Liver, Sympathetic, Shenmen, Endocrine; Body points: RN12, ST25, RN6, ST36 (bilaterally)	AA: 3-5 times daily; AP: 2-3 times daily	1 week	40	63.22 ±13.71	Routine care	40	Before care, 7 days after care	Self-Rating Anxiety Scale (SAS); Numerical Rating Scale (NRS); Pain Frequency Rating Scale	None	The SAS score showed a significant decrease, with anxiety levels markedly alleviated; the NRS pain score decreased significantly, and pain intensity was substantially reduced; the pain frequency score dropped significantly, and the occurrence of pain episodes decreased markedly.	[178]
CG	CN	SC	47.61 ±7.65	AC + WM	Acupuncture points for warm needle therapy: EX-	WA: QOD,	8 wee	60	47.69 ±7.71	WA: as protocol	60	Before treatment, 2 months after	Gastrointestinal Symptom Rating		The total effective rate in the treatment group was 93.33%; gastroscopy scores	[156]

					HN1, DU20, RN12, RN4, SP15, RN6, bilateral ST36, ST44, PC6, SP4, SP6; Acupuncture points for circular rotation technique: Right ST36, Left SP3, Left LR3	30 min; AC: QOD, 30 min	ks					treatment	Scale (GSRS)	showed a significant decrease (P<0.05); serum gastrointestinal hormones: MTL and GAS levels significantly increased, while VIP levels significantly decreased (all P<0.05); Hp conversion rate to negative was 79.24%.		
CG	CN	SC	33.56 ±10.06	AA + Mox + WM	RN12, RN4	AA: QD; Mox: QW; WM: BI	4 weeks	50	33.32 ±10.15	WM: IV BID, 14d	50	Before treatment, 14 days after treatment, 3-6 months follow-up	Numerical Pain Rating Scale	Overall efficacy rate: 96.0% (P < 0.05);	No specific adverse reactions were mentioned in the	[211]

						D										literature.	
CG	CN		70.79±2.45；	TCM + AA	ST25,RN6,RN12,ST36,SP9,SP6,BL21	TCM: QD, BID; AA: QD, 4h	4 weeks	50	70.79±2.45	WM: BID	50	Before treatment, after treatment	TCMSS	Gastric mucosal tissue changes, hemoglobin, platelets, and related inflammatory markers, HP	The treatment demonstrated a 96% efficacy rate, with reductions in TCMSS, decreased gastric mucosal pathology scores [atrophy grade, intestinal metaplasia (IM), and dysplasia (Dys)], increased hemoglobin (Hb) and platelet (PLT) counts, and decreased levels of inflammatory markers in serum [C-reactive protein (CRP), IL-6, and TNF-α) in serum, P<0.05.		[176]
CG	CN	SC	24~71,	TCM + AA	BL21,BL23,ST36,RN12,RN8	TCM: BID, 100 ml;	2 weeks	60		WM: BID, 2w	60	Before treatment, after treatment	Guidelines for Clinical Research on New Traditiona	None	An efficacy rate of 90% was achieved, with a significant reduction in the clinical main symptom score (P < 0.05).		[212]

						AA: QD, 3h							l Chinese Medicine Drugs				
CG	CN	SC	52.5 ±9.6	ACE + AC	ACE: RN12, BL18, BL19, BL21, BL21; Needle-knife release: T5-T10 articular process joint capsule, T7-T12 transverse process joint capsule	AC: QW, 2x; ACE: once after 7d	3 weeks	57	53.7 ±9.8	WM: BID+TID+QID, 14d	55	Before treatment, after treatment		Gastric electrical rhythm, gastric electrical power, gastroscopy follow-up results	Electrical rhythm and gastric electrical power both decreased (P<0.05);	No specific adverse reactions were mentioned in the literature.	[177]
CG	CN	SC	51.5 ±9.3	ACE + AC	ACE: RN12, BL18, BL19, BL21; Needle-knife release: T_5-T_{10} articular process joint capsule, T_7-T_{12} transverse	AC: Q2W, 2x; ACE: once	3 weeks	59	51.7 ±9.5	WM: QID+TID+BID, 14d	57	Before treatment, after treatment		Serum EGF levels, serum PGE_2 levels, clinical efficacy	Serum EGF and PGE_2 levels were significantly elevated (P<0.01).	No specific adverse reactions were menti	[213]

	process joint capsule	afte r 7d	oned in the literat ure.

Disease Corresponding Abbreviations: Chronic Gastritis (CG)， Chronic Non-Atrophic Gastritis (CNAG)， Chronic Atrophic Gastritis (CAG)， . China: CN, Single-center: SC, Multi-center: MC, QD: Once a day, BID: Twice a day, TID: Three times a day, QID: Four times a day, QOD: Every other day, Q2W: Every two weeks, TIW: Three times a week, d1-x: for x consecutive days, xd: x days, QW: Once a week
